# Association of Duration of Methadone or Buprenorphine Use During Pregnancy With Risk of Nonfatal Drug Overdose Among Pregnant Persons With Opioid Use Disorder in the US

**DOI:** 10.1001/jamanetworkopen.2022.7964

**Published:** 2022-04-19

**Authors:** Marian Jarlenski, Qingwen Chen, Alice Gao, Scott D. Rothenberger, Elizabeth E. Krans

**Affiliations:** 1 Department of Health Policy and Management, School of Public Health, University of Pittsburgh, Pittsburgh; 2 Center for Innovative Research on Gender Health Equity, University of Pittsburgh, Pittsburgh; 3 Division of General Internal Medicine, Department of Medicine, University of Pittsburgh, Pittsburgh; 4 Department of Obstetrics, Gynecology, and Reproductive Sciences, University of Pittsburgh, Pittsburgh; 5 Magee-Womens Research Institute, Pittsburgh, Pennsylvania

## Abstract

This cohort study evaluates the association of the duration of methadone or buprenorphine use during pregnancy and the risk of nonfatal drug overdose among pregnant persons with opioid use disorder in the US.

## Introduction

Opioid use disorder (OUD) is associated with morbidity and mortality during pregnancy and post partum,^1^ and emerging evidence suggests that drug-related deaths are a leading cause of mortality.^2^ Although pregnant persons with OUD are at high risk of drug overdose events during pregnancy and postpartum, medication for OUD (MOUD) may mitigate adverse outcomes by reducing illicit drug use and facilitating engagement with health care professionals to address co-occurring chronic conditions. Our objective was to quantify the association of the duration of MOUD use during pregnancy and the risk of nonfatal overdose in pregnancy among pregnant persons with OUD in the US.

## Methods

This retrospective cohort study was approved by the University of Pittsburgh Institutional Review Board. Informed consent was waived because deidentified national administrative data for commercially insured enrollees were used. The study followed the STROBE reporting guideline.

Using data from the Optum Clinformatics Data Mart database (version 8.1), we identified deliveries among female enrollees 15 to 44 years of age who had a live birth between January 2011 and December 2019 and were diagnosed with OUD in pregnancy. Delivery was the index date, and enrollees were followed back to the starting date of 12 weeks before the estimated date of conception. The exposure was (1) a time-varying, continuous measure of duration of MOUD use during pregnancy or (2) the period from conception to the preceding day before the overdose event occurring before delivery. MOUD use was measured as the total days’ supply of buprenorphine prescriptions or the total number of days for which methadone was dispensed. MOUD use was then aggregated to the duration of use in weeks.

The outcome was a repeated binary measure of nonfatal overdose, based on opioid-poisoning diagnoses occurring at any time during pregnancy.^3^ We modeled the relative risk (RR) of outcomes using generalized estimating equations with a Poisson regression model. Baseline covariates included continuous measures of age at conception, self-reported race and ethnicity (Asian, Hispanic, non-Hispanic Black, non-Hispanic White, and unknown), and nonopioid substance use disorders (alcohol, tobacco, amphetamines, cannabis, cocaine, or other). Race and ethnicity data were included in the analysis because past research has demonstrated racial discrimination in the provision of MOUD in pregnancy.^4,5^ We also measured the number of other clinical comorbidities, including anemia, asthma, hypertension, cardiovascular disease, kidney disease, mental health conditions, preexisting or gestational diabetes, and HIV or hepatitis C virus infection.^6^ Additional details are available in the Supplement.

## Results

This study included 2072 deliveries among 1999 female enrollees with OUD in pregnancy. A total of 1440 individuals (69.5%) had no observed MOUD use in pregnancy. Among the 632 pregnant persons (30.5%) with MOUD use, the median duration was 25.4 (IQR, 9.3-38.1) weeks (Table).

**Table.** Characteristics, Co-occurring Medical Comorbidities, and Outcomes Among Pregnant Persons With Opioid Use Disorder^a^

Variable	Value
No. of pregnancies	2072
Age at conception, y, mean (SD)	28.0 (6.0)
Race and ethnicity^b^	
Asian	31 (1.6)
Hispanic	174 (8.7)
Non-Hispanic Black	148 (7.4)
Non-Hispanic White	1550 (77.5)
Unknown/missing	96 (4.8)
Any MOUD use before conception	434 (21.0)
Comorbidities	
Any co-occurring nonopioid substance use disorder^c^	441 (21.3)
No. of medical comorbidities^d^	
0	593 (28.6)
1 to 2	1257 (60.7)
≥3	222 (10.7)
MOUD use in pregnancy (exposure)	
None	1440 (69.5)
Any	632 (30.5)
MOUD, median (IQR), wk	25.4 (9.3-38.1)
Outcome	
Any overdose	20 (1.0)
No. of overdoses	22

^a^ Values are presented as number (%) unless indicated otherwise.

^b^ The values sum to 1999 patients rather than the total number of pregnancies. Unknown/missing race includes both individuals who were not asked to state their race and ethnicity and those who declined to disclose it.

^c^ This variable includes any diagnosis of the following substance use disorders at any time in pregnancy (calculated as 280 days before delivery): tobacco, opioids, alcohol, cannabis, cocaine, amphetamines, sedatives, hallucinogens, inhalants, and other psychoactive and nonpsychoactive substances.

^d^ Number of any of the following diagnoses made at any time in pregnancy: anemia, asthma, HIV, hypertension, hepatitis C virus, mental health conditions, preexisting or gestational diabetes, or heart or kidney disease in pregnancy.

Compared with individuals with no MOUD use, there was a monotonically declining RR of nonfatal overdose events given a longer duration of MOUD use (Figure). Specifically, pregnant persons with at least 10 weeks of MOUD use had a 57% reduced risk of nonfatal overdose (adjusted RR [aRR], 0.43 [95% CI, 0.19-0.94]). Individuals with at least 20 weeks of MOUD use had an 82% reduced risk of nonfatal overdose (aRR, 0.18 [95% CI, 0.04-0.89]). Pregnant persons with at least 30 weeks of MOUD use had a 92% reduced risk of nonfatal overdose (aRR, 0.08 [95% CI, 0.01-0.84]). Finally, those with continuous MOUD use throughout pregnancy (i.e., initiated before pregnancy) had a 97% reduced risk of nonfatal overdose (aRR, 0.03 [95% CI, 0.00-0.79]).

**Figure.**
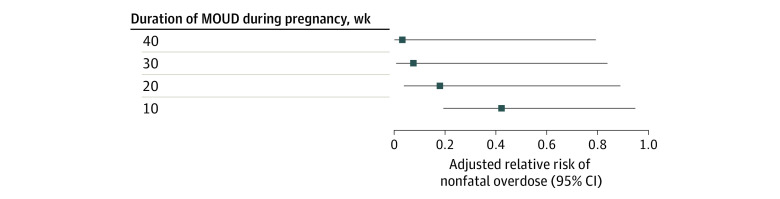
Association of the Duration of Medication for Opioid Use Disorder (MOUD) During Pregnancy and the Risk of Nonfatal Overdose Among Commercially Insured Pregnant Persons in the US Adjusted relative risks are from generalized estimating equations using a Poisson model with robust SEs. Multivariable models were controlled for patient age, race and ethnicity, number of medical comorbidities, and presence of any co-occurring nonopioid substance use disorders.

## Discussion

In this cohort study, a longer duration of MOUD use was associated with a meaningful reduction in overdose risk among pregnant persons with OUD. Study limitations include a lack of data on Medicaid-insured patients, the potential to underestimate MOUD use if patients received treatment at publicly funded clinics, and possible unmeasured confounding between MOUD use and outcomes. These results suggest that rates of nonfatal overdose in pregnant persons could be lowered substantially if MOUD was accessible throughout the entire pregnancy.

## Notes

Supplement. **eAppendix.** Statistical Modeling Methods **eFigure.** Flowchart Demonstrating the Creation of a Cohort of Commercially Insured Female Individuals With Opioid Use Disorder During Pregnancy in the US **eTable.** *International Classification of Diseases ICD-9-CM* and *ICD-10-CM* Diagnosis Codes Used to Define Exposures and Clinical Comorbidities
