# Supplementary material for: Association of Duration of Methadone or Buprenorphine Use During Pregnancy With Risk of Nonfatal Drug Overdose Among Pregnant Persons With Opioid Use Disorder in the US
Source: JAMA Netw Open. 2022 Apr 19;5(4):e227964. doi: 10.1001/jamanetworkopen.2022.7964 (PMC9020209; doi:10.1001/jamanetworkopen.2022.7964)
Supplement: Supplement. — eAppendix. Statistical Modeling Methods eFigure. Flowchart Demonstrating the Creation of a Cohort of Commercially Insured Female Individuals With Opioid Use Disorder During Pregnancy in the US eTable. International Classification of Diseases ICD-9-CM and ICD-10-CM Diagnosis Codes Used to Define Exposures and Clinical Comorbidities [file jamanetwopen-e227964-s001.pdf]

Supplementary Online Content

Jarlenski M, Chen Q, Gao A, Rothenberger SD, Krans EE. Association of duration of methadone or buprenorphine use during pregnancy with risk of nonfatal drug overdose among pregnant persons with opioid use disorder in the US. *JAMA Netw Open*. 2022;5(4):e227964. doi:10.1001/jamanetworkopen.2022.7964

eAppendix. Statistical Modeling Methods

eFigure. Flowchart Demonstrating the Creation of a Cohort of Commercially Insured Female Individuals With Opioid Use Disorder During Pregnancy in the US

eTable. *International Classification of Diseases ICD-9-CM* and *ICD-10-CM* Diagnosis Codes Used to Define Exposures and Clinical Comorbidities

This supplementary material has been provided by the authors to give readers additional information about their work.

© 2022 Jarlenski M et al. *JAMA Network Open*.

eAppendix. Statistical Modeling Methods

We modeled the relative risk of outcomes directly using a generalized estimating equation (GEE) Poisson regression model. We calculated the duration of MOUD use as the total days' supply of buprenorphine prescriptions and/or the total number of days for which methadone was dispensed. The exposure was modeled as continuous variable that measured the number of weeks' duration of MOUD from estimated date of conception to the day right before diagnosis of nonfatal overdose in pregnancy. Because nonfatal overdose events can occur multiple times during pregnancy and one episode of overdose event can last for days, we required a 5-day gap between any two diagnoses of nonfatal overdose to validate different episodes of overdose events. Nonfatal overdose events were measured through the whole pregnancy. Models included covariates based on demographic and medical factors that would likely be associated with exposures of interest and potentially causally associated with overdose. Baseline characteristics were assessed during the 12 weeks prior to the estimated date of conception. We fitted a GEE Poisson model with independent covariance structure to estimate the association between a continuous measure of weeks duration of MOUD use and risk of overdose in pregnancy, and treated patient as random-effect to account for between-subject heterogeneity. The outcome model took the following general form:

$$\log\left[\Pr\left(Y_{ijt}=1\right)\right]=\beta_0+\beta_1 MOUD_{ijt}+\beta_2 AGE_{ij}+\beta_3 R1_i+\beta_4 R2_i+\beta_9 OSUD_{ij}+\beta_{10} CC_{ijt}+b_1 PAT_i$$

Where, for the jth pregnancy of ith patient: Y_{ij}=overdose at time t during j^{th} pregnancy (binary)
$MOUD_{ijt}$=weeks of MOUD from date of conception to time $t-1$ during j^{th} pregnancy (continuous, **time-varying exposure**)
AGE_{ij}=age at date of jth conception (continuous, **baseline characteristic**)
$R1_i - R2_i$ are dummy coded variables for Race (Non-white vs. Non-Hispanic white, **baseline characteristic**)
$OSUD_{ij}$=indicator for any SUD other than OUD identified in baseline period before j^{th} pregnancy (binary, **baseline characteristic**)
CC_{ijt}=count of medical complications from date of conception to time $t-1$ during j^{th} pregnancy (continuous, 0-7, **time-varying exposure**).
PAT_i=Patient-level random effect

The Figure in the manuscript presents the adjusted relative risks of each outcome by MOUD duration in 10-week increments, with corresponding 95% confidence intervals, obtained using ESTIMATE commands within PROC GEE, using SAS version 9.4 (Cary, NC).

© 2022 Jarlenski M et al. *JAMA Network Open*.

eFigure. Flowchart Demonstrating the Creation of a Cohort of Commercially Insured Female Individuals With Opioid Use Disorder During Pregnancy in the US

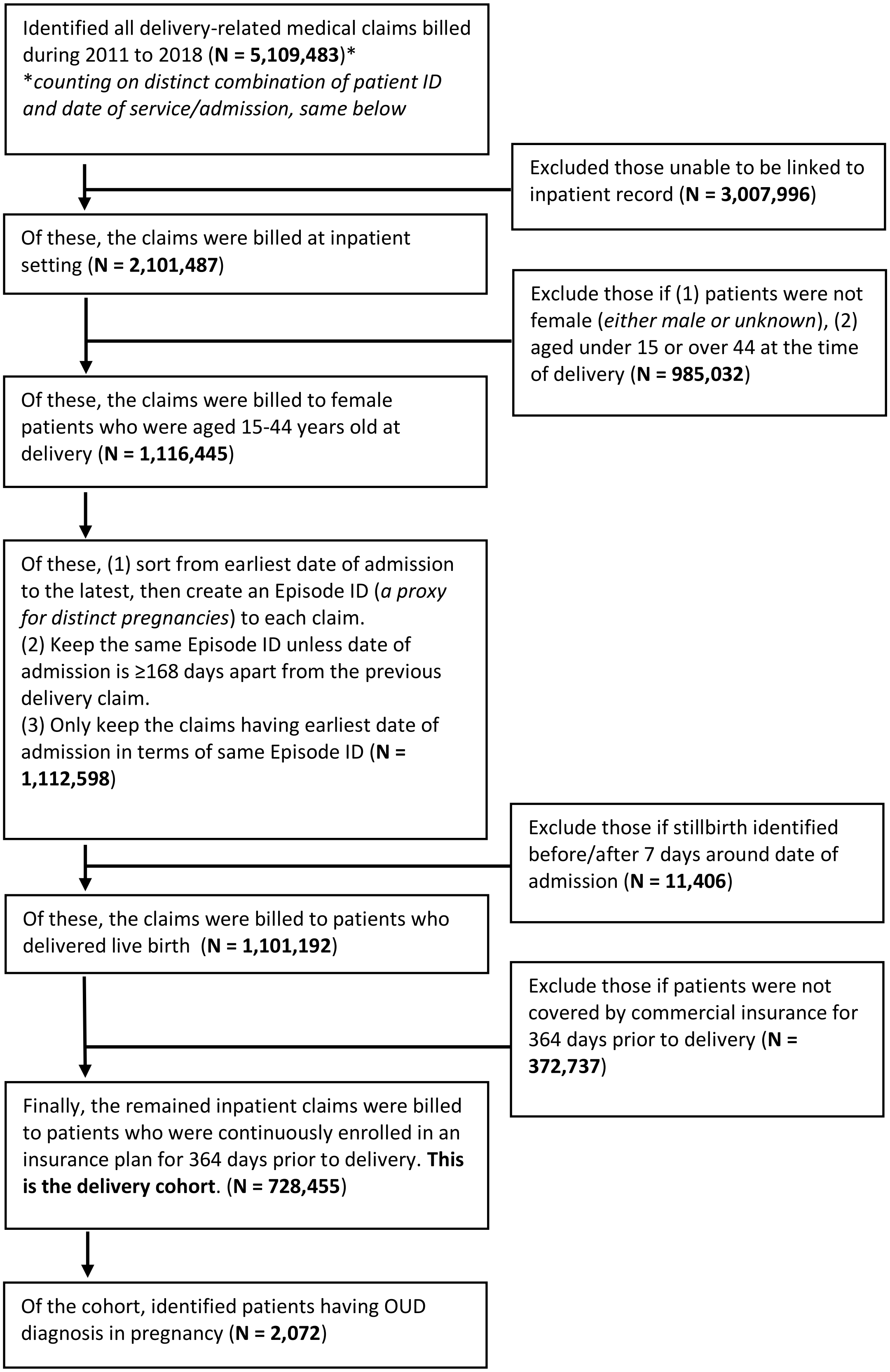

© 2022 Jarlenski M et al. *JAMA Network Open*.

eTable. *International Classification of Diseases ICD-9-CM* and *ICD-10-CM* Diagnosis Codes Used to Define Exposures and Clinical Comorbidities

	ICD-9-CM Diagnosis codes	*ICD-10-CM* Diagnosis codes
Substance use disorders		
Opioids	304.0*, 304.7*, 305.5*	F11.*
Tobacco	305.1, 649.0*, 989.84	F17.2*, O99.33*, Z72.0
Alcohol	291.*, 303.0*, 303.9*, 305.0*, 535.3*, 425.5, 357.5, 571.0, 571.1, 571.2, 571.3, 977.3, 790.3, 980.0	V11.3*, F10.*
Sedative	304.1*, 305.4*, E950.1, E950.2, 967.0, E937.0	F13.*
Cocaine	304.2*, 305.6*	F14.*
Amphetamines	304.4*, 305.7*	F15.*
Cannabis	304.3*, 305.2*	F12.*
Clinical comorbidities		
HIV/AIDS infection	042, 079.53, V08	O98.7*, B20, Z21
HCV infection	07041, 07044, 07051, 07054, V0262, 07070, 07071	B18.2, B19.2*, B17.1*
Anemia	648.21, 648.24, 285.9, 281.1, 281.2, 281.4, 281.8, 281.9, 280.1, 280.8, 280.9	O99.02, O90.81, D64.9, D53.0, D53.8, D53.9, D50.8, D50.9, D51.1, D51.2, D51.3, D51.8, D51.9, D52.*
Mental health condition (depression, anxiety, schizophrenia, bipolar disorder, suicidal ideation, suicide attempt)	295.*, 296.*, 297.*, 300.*, 301.*, 311, V62.84, E958.9	F20.*, F21.*, F22.*, F23.*, F24.*, F25.*, F26.*, F27.*, F28.*, F29.*, F30.*, F31.*, F32.*, F33.*, F34.*, F35.*, F36.*, F37.*, F38.*, F39.*, F40.*, F41.*, F42.*, F43.*, F44.*, F45.*, F46.*, F47.*, F48.*, R45.851, T91.91*
Renal disease in pregnancy	646.2*	O26.83
Heart disease in pregnancy	425.*, 648.5*, 648.6*	I42.*, O93.3*, O99.41*
Pre-existing diabetes	250.00, 250.01	O24.8*, O24.9*
Gestational diabetes	648.0*, 648.8*	O24.4*
Hypertension	642.0*, 642.1*, 642.2*, 642.3*, 642.9*	O10.0*, O26.5*, O13.9
Asthma	493.0*, 493.1*, 493.2*, 493.9*	J45.*
Thyroid disorder	648.1*	O99.28*

* Indicates any digit in *nth* position

Notes: *ICD-10-CM* code O99.320 is not used because it indicates substance use complicating pregnancy but does not specify which substance. *ICD-10-CM* code Z79.891 is not used to identify opioid use disorders because it indicates methadone administration and not opioid use.

© 2022 Jarlenski M et al. *JAMA Network Open*.
